# Music perception and speech intelligibility in noise performance by Italian-speaking cochlear implant users

**DOI:** 10.1007/s00405-021-07103-x

**Published:** 2021-10-01

**Authors:** Hilal Dincer D’Alessandro, Patrick J. Boyle, Ginevra Portanova, Patrizia Mancini

**Affiliations:** 1 Department of Audiology, Faculty of Health Sciences, Hacettepe University, Ankara, Turkey; 2 Department of Experimental Psychology, Cambridge University, Cambridge, UK; 3 European Research Center, Advanced Bionics GmbH, Hannover, Germany; 4 Department of Sense Organs, Sapienza University of Rome, Viale dell’Università 31, 00161 Rome, Italy

**Keywords:** Cochlear implant, Speech perception, Speech intelligibility in noise, Music perception

## Abstract

**Objective:**

The goal of this study was to investigate the performance correlations between music perception and speech intelligibility in noise by Italian-speaking cochlear implant (CI) users.

**Materials and methods:**

Twenty postlingually deafened adults with unilateral CIs (mean age 65 years, range 46–92 years) were tested with a music quality questionnaire using three passages of music from Classical Music, Jazz, and Soul. Speech recognition in noise was assessed using two newly developed adaptive tests in Italian: The Sentence Test with Adaptive Randomized Roving levels (STARR) and Matrix tests.

**Results:**

Median quality ratings for Classical, Jazz and Soul music were 63%, 58% and 58%, respectively. Median SRTs for the STARR and Matrix tests were 14.3 dB and 7.6 dB, respectively. STARR performance was significantly correlated with Classical music ratings (*r*_s_ = − 0.49, *p* = 0.029), whereas Matrix performance was significantly correlated with both Classical (*r*_s_ = − 0.48, *p* = 0.031) and Jazz music ratings (*r*_s_ = − 0.56, *p* = 0.011).

**Conclusion:**

Speech with competitive noise and music are naturally present in everyday listening environments. Recent speech perception tests based on an adaptive paradigm and sentence materials in relation with music quality measures might be representative of everyday performance in CI users. The present data contribute to cross-language studies and suggest that improving music perception in CI users may yield everyday benefit in speech perception in noise and may hence enhance the quality of listening for CI users.

## Introduction

The primary goal of cochlear implant (CI) technology has been to restore functional hearing and speech perception in people with bilateral severe-to-profound sensorineural hearing loss. Indeed, numerous studies have proven that most postlingually deafened CI users are able to achieve hearing thresholds that allow them to show very good understanding of open-set sentences presented in quiet [1]. Likewise, most congenitally deaf children, implanted within sensitive periods, acquire spoken language skills at the level of their normal hearing (NH) peers [1, 2]. However, CI users’ performance tends to deteriorate significantly for more complex tasks, such as music perception and speech intelligibility in noise [3, 4]. Even the performance of the best CI performers such as bilateral or bimodal listeners is still considerably poorer than that of NH people [5, 6]. Indeed, recent efforts in CI technology focus on improving CI users’ performance in everyday listening environments, where speech with competitive noise and music are usually present. The principal focus has been to improve CIs’ technical constraints in dynamic range and in the resolution of spectro-temporal content of acoustic signals. These are known as key factors resulting in significant performance deterioration for complex but typical everyday listening activities [7].

Music and spoken language have several characteristics in common and share several terms, such as duration, tempo, rhythm, and pitch [3]. In fact, music, like spoken language, appears to be an innate skill and to appear almost in all cultures throughout history [8]. Both are characterized by varying intensity and frequency over time; however, music typically spans a wider spectrum, dynamic range and spectro-temporal content. Musical information is often presented to the listener simultaneously; hence, perception of music often requires simultaneous processing of rhythm, pitch, timbre, and melodies produced by various instruments. Pitch cues in particular, linked to fundamental frequency (F0) perception and fine structure processing of acoustic signals, are of utmost importance for music perception and speech intelligibility in the presence of noise [9]. Despite individual differences, most CI users are able to discriminate rhythmic patterns as well as NH people [3, 10, 11]. However, the pitch and timbre perception skills of CI users are significantly poorer than those of NH listeners, or hearing aid users with a similar degree of hearing loss [3, 12]. As a result, outcomes of melody perception without rhythmic cues deteriorate dramatically in CI users as does speech perception in noise [3].

Music contributes significantly to auditory learning because of the overlap in auditory pathways and brain networks that process linguistic and musical cues. Acoustically rich musical stimuli activate the bilateral network in the brain related to auditory stream analysis, semantic and emotional processing, motor functions, attention and working memory [3, 13, 14]. Playing or practicing a music instrument is thought to tune the auditory system by enhancing auditory sensory encoding, top-down processing, and the cross-modal integration that contributes significantly to signal-in-noise perception [11, 15]. Music training encourages more concentrated listening, because the sensitivity required for music processing is higher than that of speech without competition, and greater attention is required to focus on the details in musical sounds and to integrate different concurrently occurring instruments or melodies [14]. Indeed, some evidence comes from studies that compare NH musicians and non-musicians’ performance. These studies reveal significant musician advantages for higher level auditory functions, such as perception of signal-in-noise and spectrally degraded stimuli simulating CI signal processing [14–17]. Such findings are thought to be promising for studies in CI users, in terms of similarities in performance for music perception and speech understanding in noise. Indeed, some CI studies observe positive effects for music training especially for speech perception in noise [3, 7]. Such effects might be partially explained by the improvements in some aspects of cognitive–linguistic functioning, for example auditory attention and phonological processing [13, 18]. On the other hand, fine structure and pitch perception skills improved through music training might be transferred to speech perception, considering their importance especially for speech intelligibility in noise [7, 10].

Research relating speech intelligibility in the presence of noise to music perception for CI users is still very limited. Rather, the majority of the existing studies focus on speech understanding in quiet [20]. To our knowledge, in the literature, there are only two studies specifically investigating music perception in relation to speech intelligibility in noise, and their findings are not concordant [19, 20]. The first study conducted by Won et al. [19] reports that the pitch direction discrimination skills as well as melody and timbre recognition scores in Cl users are significantly correlated with Speech Reception Thresholds (SRTs) in noise for a closed-set spondee identification task. The second one, a very recent study by Fowler et al. [20] conducted in 10 NH adults and a group of 10 mostly prelingually deafened adult CI users shows that better music perception skills are related to better speech intelligibility in noise scores only for combined data of NH and CI participants, whereas the statistical analysis in CI group alone does not reveal any significant correlations between the two performances. On the other hand, the common characteristics of these studies are the use of perceptual accuracy measures for music perception and the use of English language speech material. However, CI users’ real-world musical experience, or personal opinions and appraisals of music quality may not represent only their abilities in perceptual accuracy [11]. In addition, studies based on languages other than English may enlighten language-specific findings and allow cross-language comparisons of music and speech perception skills for CI users. Moreover, some recent speech perception tests based on an adaptive paradigm, or the use of sentence materials as opposed to single isolated words of a closed-set task, might be more representative of everyday performance in CI users. Thus, the current study aims to investigate the performance correlations between music perception and speech intelligibility in noise by Italian-speaking CI users. Music perception was assessed through a music quality questionnaire, using three passages of music from Classical Music, Jazz, and Soul [21, 22]. Speech recognition in noise was assessed using two recently developed adaptive tests in Italian: the STARR [23] and Matrix tests [24]. The STARR test was developed to mimic real life listening conditions, where speech and noise levels vary together [23, 25]. On the other hand, the Matrix test aims to yield a high reliability across several languages, including Italian, for speech perception assessment, where the use of adaptive noise is also available [24, 26].

## Materials and methods

### Participants

The study participants were 20 postlingually deafened adult CI users who were consistent unilateral CI wearers (7 female and 13 male). Demographic information and audiological data for individual participants are given in Table 1. The mean age was 65 years (range 46–92 years, SD = 12.7). The mean duration of deafness was 58 months (range 3–420 months, SD = 117.2). All participants had a minimum CI experience of 6 months (mean = 61 months, range 6–177 months, SD = 59.7). Eight participants were implanted with Advanced Bionics (Valencia, USA) devices whilst twelve participants were implanted with Med-El (Innsbruck, Austria) devices. Advanced Bionics devices were 90 K implants fitted with HiRes-S (*n* = 3) or HiRes Optima-S (*n* = 5). Med-El devices were Concerto implants fitted with FS4 (*n* = 6) or FS4-p (*n* = 6)]. The participants showed no degree of low frequency residual hearing in the implanted/contralateral ear. The mean CI pure tone threshold in the sound field was 32.2 dB HL (range 20.0 to 40 dB HL, SD = 6.4) for octave frequencies between 125 and 8000 Hz. The mean word recognition score for listening in quiet was 68.5% (range 20–100%, SD = 30.5). The present study was approved by the Local Ethical Committee (code: n. 259/2020) and written consent was obtained from all participants. This study was carried out in accordance with the ethical requirements of the Helsinki Declarations, the Epidemiological Good Practice Guidelines of the International Conference of Harmonization, and the existing legislation in Italy.

**Table 1:** Demographic information and audiological data for individual participants

ID	CI ear	Age (years)	CI use (months)	Duration of deafness (months)	CI model/processor	Sound coding strategy	PTA (dB HL)	WRS (%)	STARR (dB)	Matrix (dB)
P1	R	63	11	12	Med-El, Sonnet	FS4	40	60	30	12.2
P2	R	71	91	12	Med-El, Sonnet	FS4	31.3	20	30	6
P3	R	76	174	360	AB, Naìda	HiRes Optima-S	37.5	80	12.1	9
P4	L	83	21	36	Med-El, Opus 2	FS4-p	40	70	30	30
P5	L	71	6	3	Med-El, Sonnet	FS4	39.4	20	30	30
P6	R	46	19	3	Med-El, Sonnet	FS4	23.1	20	30	30
P7	R	55	10	120	AB, Naìda CI Q70	HiRes Optima-S	27.5	70	1.6	2.7
P8	R	61	13	3	Med-El, Rondo	FS4	30	100	1.6	1.9
P9	L	50	25	3	Med-El, Rondo	FS4-p	40	30	30	3.5
P10	L	74	18	12	Med-El,Sonnet	FS4-p	31.9	60	4.8	21.4
P11	R	79	26	10	AB, Naìda CI Q70	HiRes Optima-S	32.5	100	10.8	12.2
P12	R	59	33	25	Med-El, Sonnet	FS4-p	20	80	30	30
P13	L	59	65	6	AB, Naìda CI Q90	HiRes-S	33.8	100	7.6	6.1
P14	L	68	177	3	AB, Harmony	HiRes-S	35.6	100	13.1	5.4
P15	R	54	107	3	AB, Naìda CIQ70	HiRes Optima-S	26.9	100	2.8	2.4
P16	R	92	152	3	AB, Harmony	HiRes Optima-S	28.1	90	10.6	4.3
P17	L	50	158	36	Med-El, Sonnet	FS4	20	90	0.8	2.4
P18	R	79	39	60	AB, Naìda CI Q70	HiRes-S	37.5	90	15.6	5
P19	R	51	40	36	Med-El, Opus 2	FS4-p	31.3	20	30	30
P20	R	67	30	420	Med-El, Sonnet	FS4-p	36.9	70	30	30

*CI* cochlear implant, *PTA* pure tone average, *WRS* Word Recognition Score in Quiet

### Procedure

All testing was carried out in a sound-proofed room. The speech and music stimuli were presented through an Acer P253-MG computer (Hscinchu City, Taiwan) and a Sony TA-FE 320R preamplifier (Tokyo, Japan) connected directly to a single Tangent EVO E5 loudspeaker (Herning, Denmark) at 0° azimuth and at 1 m distance from the listener’s head. For testing, the participants were asked to set their sound processors to a comfortable listening level. Speech recognition in quiet was performed using the disyllabic phonemically balanced word lists [27] presented at 65 dB SPL. Test orders for speech and music perception assessments were counterbalanced across participants to minimize any learning effects. Any test session, including both tonal and speech audiometry, as well as music perception assessment did not exceed 1 h.

### Assessment of speech perception in noise

#### The STARR test

The STARR test was originally developed in British English, with the aim to represent everyday listening conditions, where speech often needs to be understood in the presence of noise and can vary considerably from the levels commonly used in speech testing [25]. The Italian adaptation made use of everyday sentences, all recorded with a male voice [23]. The Italian STARR consisted of 10 test lists, each containing 15 sentences. Speech-shaped noise was used for competition. Sentences were presented in randomized order at three presentation levels (50, 65 and 80 dB SPL). There were five presentations at each level within a single test list. The number of words in each sentence ranged from 3 to 7. Each sentence consisted of three key words that were used for scoring. After presentation of a sentence, listeners were asked to repeat it as accurately as possible but were told that not every word had to be correct. For the response to a sentence to be scored as correct, at least two out of the three key words must have been repeated back correctly. The initial SNR was + 20 dB and varied adaptively following the listener’s response. Following a correct response, a more adverse SNR was used for the next sentence. Conversely, after an incorrect response, a more favorable SNR was used for the next sentence. The SNR step size started at 10 dB; dropped to 5 dB after the first reversal of the adaptive track and dropped again to 2.5 dB after a further reversal. The SNR was varied by adjusting the noise level to suit the speech level of 50, 65 or 80 dB SPL, allowing the adaption of SNR across all three speech levels. The SRT was calculated automatically by averaging the SNRs for the last nine sentences together with the SNR at which a next sentence would have been presented. The test–retest reliability was 0.7 dB for the Italian STARR test [23]. Before testing, a training list was administered to minimize any learning effects and SRTs from two test lists were averaged to provide the STARR score for each listener. While the STARR SRTs could theoretically range very widely, based on physical limitations, such as the test booth’s noise floor and the dynamic range of the amplification system, an SNR range of − 10 dB to + 30 dB was set for SRTs.

#### The matrix sentence test

The Matrix test consisted of semantically unpredictable but syntactically fixed sentences (name–verb–numeral–noun–adjective, e.g., ‘*Anna prende quattro tazze normali*’, which is Italian for ‘Anna takes four normal cups’). The Matrix test consisted of a vocabulary of 50 commonly used words of 2 to 3 syllables, from which test sentences were randomly generated (10 alternatives for each position in the sentence). The masking noise with the same long-term spectrum of the speech material was generated through 30-fold overlapping of all the sentences. The test–retest reliability was found to be 0.5 dB for the Italian Matrix test [24]. The present Matrix testing was carried out using an open-set response format. The noise level was fixed at 65 dB SPL and an SNR of 0 dB was presented initially. Each test list was composed of 30 sentences and testing was preceded by two training lists to minimize learning effects. Based on the number of correctly repeated words in the preceding sentence, the software decided the speech level for the next sentence ultimately estimating the SNR, where 50% of words were repeated correctly. Similarly to the STARR test, an SNR ceiling value of 30 dB was set for participants’ performance showing no response on the Matrix test.

### Assessment of music perception

Music perception assessment consisted of three passages of three different types of music (Classical Music, Jazz, and Soul) and of a music quality questionnaire [21, 22], introduced by Advanced Bionics in several languages, including English and Italian. Passages for Classical and Jazz music were instrumental whilst Soul music was a passage featuring a single female voice. Each passage lasted 1 min. The order of presentation was randomized between the participants who were asked to express their appreciation of various aspects of the specific passage to which they had just listened. The questionnaire consisted of 12 questions relative to the appreciation of music. The first six questions were based on evaluating clarity, pleasantness, naturalness, overall quality, boominess and tinniness of passages. This was scored on a Likert scale from 0 to 10, where higher scores revealed better perception, e.g., 0 = Extremely unclear or extremely unpleasant; 3 = Unclear or unpleasant; 7 = Clear or pleasant; 10 = extremely clear or extremely pleasant. The following three questions (7 to 9) were yes (scored as 1) or no (scored as 0) questions regarding the loudness and the rhythm of the passages. Finally, there were three more questions relative to the identification of the passages, such as the identification of music style (please see Table 2 for details of questions). Here, only the correct choices were scored as 1 and all the wrong ones as 0. For statistical analysis, the responses were converted to percentage scores with the following formula (score/total score × 100).

**Table 2:** Percentages of CI listeners with good perception for Classical music, Jazz and Soul

Questions	Classical (%)	Jazz (%)	Soul (%)
1. Clarity of passage	30	60	55
2. Pleasantness of passage	45	55	60
3. Overall quality of passage	35	45	50
4. How boomy does the passage sound?	55	70	75
5. How tinny does the passage sound?	70	70	70
6. How natural is the passage?	40	60	55
7. Were any sections of the passage uncomfortably loud?	60	20	20
8. Were any sections of the passage inaudible?	60	25	25
9. Do you think the passage had a well-defined rhtym?	30	20	35
10. Was the passage mainly: vocals/instrumental	90	95	95
11. Could you detect: a male voice/a female voice/multiple voices/no voices	70	85	90
12. What category of music would best describe the passage: Classical/Jazz/Pop/Soul/folk/other	80	45	25

For the first six questions scores equal to or greater than 7 are considered as good. For yes/no questions the positive evaluation was considered. For the last three questions the correct responses were considered

### Statistical analysis

Data analysis was carried out with the Statistical Package for Social Sciences (SPSS version 25.0, IBM Corporations, Chicago, IL, USA). Analysis showed that the data from STARR and Matrix tests were not normally distributed (*p* ≤ 0.001); hence, non-parametric statistical tests were performed. A Friedman test was carried out to compare Classical, Jazz and Soul music performance differences. Percentages of good performers were calculated based on scores ≥ 7 for Likert scale questions (1 to 6), whilst positive responses (7 to 9) or correct responses (10 to 12) were considered for the following questions. Within-subjects differences between STARR and Matrix performances were investigated using Wilcoxon Signed Ranks test. Bivariate correlations between speech and music perception performances as well as between STARR and Matrix performances were examined using the Spearman rank-order correlations. The effects of demographics such as age, duration of deafness and duration of CI experience, as well as the effects of CI pure tone thresholds on speech and music perception, were tested with the Spearman rank-order correlations. The cutoff level for statistical significance was set to 0.05 and only statistically significant correlations were reported for demographics.

## Results

Table 2 gives percentages of CI users with good perception for single questions from Classical music, Jazz and Soul. Table 3 lists percentiles for speech and music perception performance.

**Table 3:** Percentiles for speech and music perception performance

Percentiles	STARR (dB)	Matrix (dB)	Classical (%)	Jazz (%)	Soul (%)
25	5.5	3.7	52	48	45
50	14.3	7.6	63	58	58
75	30	30	71	65	64

Median quality ratings for Classical, Jazz and Soul music were 63% (range 27–92%), 58% (range 30–75%) and 58% (range 26–83%), respectively. Differences between Classical, Jazz and Soul music ratings were not statistically significant [X^2^ (2, *N* = 20) = 2.8, *p* = 0.247]. The correlations between music genres were statistically significant [(*r*_s_ = 0.76, *p* < 0.001) for Classical versus Jazz, (*r*_s_ = 0.68, *p* = 0.001) for Jazz versus Soul and (*r*_s_ = 0.60, *p* = 0.005) for Classical versus Soul music].

The median STARR test SRT was 14.3 dB SNR (range 0.8 to 30 dB). The percentage of participants with a score better than the SNR ceiling value of 30 dB was 55%. STARR performance was significantly correlated with Classical music ratings (*r*_s_ = − 0.49, *p* = 0.029). There was a tendency towards significant correlations with Jazz music ratings (*r*_s_ = − 0.44, *p* = 0.054) whilst correlations with Soul music ratings were not statistically significant (*r*_s_ = − 0.18, *p* = 0.448).

The median Matrix test SRT was 7.6 dB SNR (range 1.9 to 30 dB). The percentage of participants with a score better than the SNR ceiling value of 30 dB was 70%. Matrix performance was significantly correlated with both Classical (*r*_s_ = − 0.48, *p* = 0.031) and Jazz music ratings (*r*_s_ = − 0.56, *p* = 0.011) whilst correlations with Soul music ratings were not statistically significant (*r*_s_ = − 0.18, *p* = 0.442).

Matrix performance was significantly correlated with STARR outcomes (*r*_s_ = 0.72, *p* < 0.001) and performance differences between the tests were not statistically significant (*Z* = − 1.7, *p* = 0.084).

CI pure tone averages were significantly correlated with STARR performance (*r*_s_ = 0.45, *p* = 0.044). CI experience was significantly correlated with music perception (*r*_s_ = 0.54, *p* = 0.015 for Classical, *r*_s_ = 0.62, *p* = 0.004 for Jazz and *r*_s_ = 0.58, *p* = 0.008 for Soul music ratings).

## Discussion

Music is a natural part of everyday life, and is encountered almost everywhere, including home, kindergarten, school, office, social events such as concerts or sports competitions, or on radio or TV. Not surprisingly, music seems to exist as a culturally important acoustic phenomenon and as a common mode of communication in almost all known cultures. It supports mental wellbeing, brings people together via collective memories, promotes lifelong social harmony, and elicits emotional meaning. Indeed, as we all have had to experience over the past year due to the COVID-19 pandemic, the inability to participate in social–musical activities such as going to concerts or singing with friends may negatively affect quality of life and lead to feelings of loneliness or isolation, about which people with hearing impairment very often complain [28–30]. Similarly, background noise is usually inevitable for everyday listening environments, such as parks, restaurants, schools and offices. This fact leads to communication difficulties for CI users in daily life and negatively affects their quality of life [30]. Indeed, both music and speech perception in noise share the same limitations of today’s CI technology, in particular for the transmission of spectro-temporal cues of acoustic signals.

In recent years, there has been a growing interest in the perception of music and speech in noise by CI users. An overview of studies on music perception in CI users shows that most of them focus on evaluating perceptual accuracy for rhythm, pitch, timbre, and melody perception. Despite individual differences in performance, most CI users can distinguish rhythmic patterns and may even show rhythm perception skills similar to people with normal hearing [3, 10, 11]. However, pitch and timbre perception performance of CI users are significantly poorer than both NH individuals and hearing aid users with similar degrees of hearing loss [3, 12, 31]. Therefore, in CI users, outcomes for melody perception without rhythmic and verbal cues deteriorate significantly due to insufficient transmission of pitch and timbre information [3, 10]. Pitch and timbre are known as the basic elements of spoken language and music. On the other hand, these elements are thought to be the source of pleasure and other emotions when listening to music. Indeed, another point of interest in the field is how much CI users enjoy listening to music [3, 10, 11, 31]. However, studies for music appreciation in CI users are still much more limited compared to studies on perceptual accuracy such as rhythm, timbre and melody discrimination or recognition skills. Despite an improvement in the behavior and duration of music listening after implantation, enjoyment is rarely observed at the level of NH people in CI users. On the other hand, their listening habits, preferences, and attitudes are very heterogenous. The majority of postlingually deafened CI users state that their hearing loss negatively affects their musical experience [3]. The present study is consistent with such findings in terms of reflecting several participants who do not find music sounds clear, pleasant, or natural and complain about the overall quality of musical passages. Despite the absence of statistical differences between music genres, considerably poorer music quality ratings were observed for the Classical music, which might be a more challenging listening task due to its symphonic, instrumental content, involving more complex characteristics and a wider dynamic range. Indeed, the percentage of participants who reported inaudible or uncomfortably loud sections for the Classical music passage (60%) was remarkably higher than for the Jazz (20%) or Soul music (25%). Surprisingly, the percentage of participants who stated that the passages had a well-defined rhythm is very low (⁓30%) for all music genres, although it is a well-known fact that perceptual findings for rhythm discrimination in postlingually deafened CI users are comparable to those of NH listeners [3, 10, 11]. Such results might be partly due to differences between perceptual accuracy and appraisal. Music appraisal does not only reflect perceptual accuracy, but is also dependent on personal, situational, and emotional factors. Rather than perceptual accuracy, it is the music listening and appreciation that represents a functional estimation of an individual’s real-world experience with music [11]. On the other hand, musical culture may have significant effects on music perception in postlingually deafened CI users. Such possible effects have not been evaluated in this study, but they might be very interesting to consider in future studies.

As reported above, several studies show that the majority of postlingually deafened CI users complain that they no longer enjoy listening to music. Conversely, prelingually deafened children start listening to music with pleasure after cochlear implantation. In fact, pediatric CI users enjoy musical activities such as singing, dancing or playing an instrument [3, 12]. Indeed, to get their first musical experience with a CI might be an advantage for children [3, 32]. However, postlingually deafened individuals have a normal representation of music stored in their memory. Hence, they might be comparing the music they listen to through CI with their previous experience and become frustrated. Such arguments might be further supported by our present findings, showing significant correlations between duration of CI experience and music perception. This may suggest that postlingually deafened CI users may need a longer time to adopt and appreciate musical sounds that they hear with their implant. This is like what happens for other complex listening situations, such as speech perception in noise, that continue to improve even after 6 months of CI experience [33]. In this sense, some evidence comes from previous studies by Looi and She [34] and Moran et al. [11] that observe a correlation between the time spent listening to music and enjoyment of music. Moreover, CI users with better speech intelligibility in noise scores report higher levels of music listening and enjoyment. Such findings may emphasize the importance of encouraging and motivating CI users’ music listening in the post-operative rehabilitation process.

Speech intelligibility performance using the STARR and Matrix tests has been already investigated both in adult NH and CI populations. The average SRTs in Italian-speaking NH listeners have been found to be − 8.4 dB for the STARR test [23] and − 7.3 dB for the Matrix test [24]. However, outcomes for both STARR and Matrix tests in CI users are shown to be significantly poorer (⁓ 15 dB poorer) than NH people [5, 24]. The present findings are in line with such outcomes and reveal that both tests provide insights into the communication difficulties that CI users face in the presence of noise. Even the best CI performers from the present sample are not able to achieve STARR or Matrix performance similar to that reported in NH people. Several participants show SRTs of 30 dB SNR meaning that they could simply not understand speech, either essentially in quiet, or certainly when noise was present. The tests’ outcomes are strongly correlated, and performance differences are not statistically significant, although the central tendency from the present sample seems considerably better for the Matrix test. The differences in scores between the two tests might be mainly due to their different characteristics, such as syntactic and semantic structure. Despite difficulties of semantic unpredictability of Matrix sentences, the closed set nature of the test may help CI users to achieve a better score. Conversely, the STARR test uses everyday familiar sentences that are semantically meaningful and allow them to benefit from the predictability of the speech material. On the other hand, the tests differ in the adaptive paradigms as well. Here, the Matrix test is based on a fixed noise level with a varying speech level, whereas the STARR test uses an adaptive paradigm that will deliberately rove the speech material over a 30 dB range, exercising the sound processor’s automatic gain control and compression functions. This characteristic of the STARR makes the test more challenging but is believed to provide a better reflection of the real-life performance in CI users. In particular, low-level speech presentation has been found as the determining factor for poorer performance in CI users. Indeed, very high STARR SRTs illuminate CI listeners’ difficulties in understanding low-level speech even in the absence of detectable competing noise [23–26]. Such results are further supported by the present significant STARR correlations with CI pure tone averages highlighting the test’s sensitivity to low-level speech in CI users. As reported above, such inaudibility or loudness problems resulting in perceptual difficulties are also observed for music passages, especially for Classical music. This might be mainly due to the sound processing principles of present-day CI systems being based on mapping the wide dynamic range of acoustic signals into the limited electrical dynamic range. A further consideration is the transmission of low-to-high-frequency information. Acoustic signals are filtered into bands of frequencies using a bank of bandpass filters and the output of each bandpass channel is assigned to a single electrode, from apical-to-basal electrodes, to mimic the tonotopic organization of the normal cochlea but usually with a frequency-to-place mismatch. Thus, envelope variations in the acoustic signals are transmitted whilst fine structure information is largely lost in the process of CI sound processing [1]. Such limitations in spectro-temporal encoding do not allow CI users to perceive fine details in acoustic signals that are of the utmost importance for music perception and speech intelligibility in noise [9]. Indeed, statistically significant correlations between music quality ratings and speech intelligibility in noise are observed only with Classical music for STARR scores, and with Classical and Jazz music for Matrix scores but not with more “simple” Soul music passage based on a single female voice. Conversely, Classical and Jazz music passages are instrumental and involve a wider dynamic range as well as more complex characteristics (e.g., a substantial increase in the number of auditory signals lead by a greater number of harmonics) [35]. CI users usually comment that music may sound “noisy”, especially if played by large instrument ensembles. Due to limited spectro-temporal resolution, CI users have difficulties to segregate and stream competing sound sources. Indeed, concurrent presence of various instruments and melodies in music may even serve as competitive “noise” for CI users. Likewise, CI users seem not to be able to listen in the dips, which provides the ability to distinguish whether a signal in a fluctuating background is the target speech or the noise [36].

In conclusion, the present study’s correlational findings show the likeliness that both music and speech perception skills are constrained by the peripheral and higher level auditory processing sensitivity in CI users. However, it should be also noted that significant correlations from present data may not necessarily reflect a causative mechanism [37]. Nevertheless, these data suggest that improving music perception in CI users may yield clinical and everyday benefit in speech perception in noise and may enhance the quality of life for CI users. Further research in larger CI populations may allow performance comparisons based on the effects of speech coding strategies or stimulation modes such as unilateral, bilateral and bimodal implantation. Moreover, the use of quality of life measures might be beneficial to monitor improvements in everyday performance and quality of life.

## References

[1] Wilson BS, Dorman MF (2008). Cochlear implants: current designs and future possibilities. J Rehabil Res Dev.

[2] Mancini P, Dincer D’Alessandro H, Guerzoni L, Cuda D, Ruoppolo G, Musacchio A, Di Mario A, De Seta E, Bosco E, Nicastri M (2015). Adequate formal language performance in unilateral cochlear implanted children: is it indicative of complete recovery in all linguistic domains? Insights from referential communication. Int J Pediatr Otorhinolaryngol.

[3] Looi V, Gfeller K, Driscoll V (2012). Music appreciation and training for cochlear implant recipients: a review. Semin Hear.

[4] Gfeller K, Guthe E, Driscoll V, Brown CJ (2015). A preliminary report of music-based training for adult cochlear implant users: rationales and development. Cochlear Implants Int.

[5] Dincer D’Alessandro H, Ballantyne D, Boyle PJ, De Seta E, DeVincentiis M, Mancini P (2018). Temporal fine structure processing, pitch, and speech perception in adult cochlear implant recipients. Ear Hear.

[6] Parkinson AJ, Rubinstein JT, Drennan WR, Dodson C, Nie K (2019). Hybrid music perception outcomes: implications for melody and timbre recognition in cochlear implant recipients. Otol Neurotol.

[7] Firestone GM, McGuire K, Liang C, Zhang N, Blankenship CM, Xiang J, Zhang F (2020). A preliminary study of the effects of attentive music listening on cochlear implant users’ speech perception, quality of life, and behavioral and objective measures of frequency change detection. Front Hum Neurosci.

[8] Ellis P (2004). Vibroacoustic sound therapy: case studies with children with profound and multiple learning difficulties and the elderly in long-term residential care. Stud Health Technol Inform.

[9] Moore BJC (2008). The role of temporal fine structure processing in pitch perception, masking and speech perception for normal-hearing and hearing-impaired people. J Assoc Res Otolaryngol.

[10] Limb CJ, Rubinstein JT (2012). Current research on music perception in cochlear implant users. Otolaryngol Clin North Am.

[11] Moran M, Rousset A, Looi V (2016). Music appreciation and music listening in prelingual and postlingually deaf adult cochlear implant recipients. Int J Audiol.

[12] Gfeller K, Driscoll V, Schwalje A (2019). Beyond technology: the interaction of perceptual accuracy and experiential factors in pediatric music engagement. Otol Neurotol.

[13] Shahin AJ (2011). Neurophysiological influence of musical training on speech perception. Front Psychol.

[14] Patel AD (2014). Can nonlinguistic musical training change the way the brain processes speech? The expanded OPERA hypothesis. Hear Res.

[15] Parbery-Clark A, Skoe E, Lam C, Kraus N (2009). Musician enhancement for speech-in-noise. Ear Hear.

[16] Fuller CD, Galvin JJ, Maat B, Free RH, Baskent D (2014). The musician effect: does it persist under degraded pitch conditions of cochlear implant simulations?. Front Neurosci.

[17] Zhang F, Roland C, Rasul D, Cahn S, Liang C, Valencia G (2019). Comparing musicians and non-musicians in signal-in-noise perception. Int J Audiol.

[18] Musacchia G, Strait D, Kraus N (2008). Relationships between behavior, brainstem and cortical encoding of seen and heard speech in musicians and non-musicians. Hear Res.

[19] Won JH, Drennan WR, Kang RS, Rubinstein JT (2010). Psychoacoustic abilities associated with music perception in cochlear implant users. Ear Hear.

[20] Fowler SL, Calhoun H, Warner-Czyz AD (2021). Music perception and speech-in-noise skills of typical hearing and cochlear implant listeners. Am J Audiol.

[21] Dincer D’Alessandro H (2021). Speech and music perception in cochlear implant users. Turk Klin J Health Sci.

[22] Filipo R, Ballantyne D, Mancini P, D’Elia C (2007) Music perception in adult HiRes 120 users. Advanced bionics auditory research bulletin 2007. AB studies and research|advanced bionics. AB Auditory Research Bulletin 2007 (advancedbionics.com). https://advancedbionics.com/content/dam/advancedbionics/Documents/Global/en_ce/Professional/AB-Studies-and-Research/Auditory-Research-Bulletins/AB_Auditory_Research_Bulletin_2007_Biennial_Edition.pdf. Accessed 14 May 2021

[23] Dincer D’Alessandro H, Ballantyne D, De Seta E, Musacchio A, Mancini P (2016). Adaptation of the STARR test for adult Italian population: a speech test for a realistic estimate in real-life listening conditions. Int J Audiol.

[24] Puglisi GE, Warzybok A, Hochmuth S, Visentin C, Astolfi A, Prodi N, Kollmeier B (2015). An Italian matrix sentence test for the evaluation of speech intelligibility in noise. Int J Audiol.

[25] Boyle PJ, Nunn TB, O’Connor AF, Moore BCJ (2013). STARR: a speech test for evaluation of the effectiveness of auditory prostheses under realistic conditions. Ear Hear.

[26] Kollmeier B, Warzybok A, Hochmuth S, Zokoll MA, Uslar V, Brand T, Wagener KC (2015). The multilingual matrix test: principles, applications, and comparison across languages: a review. Int J Audiol.

[27] Turrini M, Cutugno F, Maturi P, Prosser S, Leoni FA, Arslan E (1993). Bisyllabic words for speech audiometry: a new Italian material. Acta Otorhinolaryngol Ital.

[28] Mick P, Kawachi I, Lin FR (2014). The association between hearing loss and social isolation in older adults. Otolaryngol Head Neck Surg.

[29] MacDonald RAR (2013). Music, health, and well-being: a review. Int J Qual Stud Health Well-being.

[30] Dritsakis G, van Besouw RM, O’Meara A (2017). Impact of music on the quality of life of cochlear implant users: a focus group study. Cochlear Implants Int.

[31] McDermott HJ (2004). Music perception with cochlear implants: a review. Trends Amplif.

[32] Driscoll V, Gfeller K, Tan X, See RL, Cheng H, Kanemitsu M (2015). Family involvement in music impacts participation of children with cochlear implants in music education and music activities. Cochlear Implants Int.

[33] Lenarz M, Sönmez H, Joseph G, Büchner A, Lenarz T (2012). Cochlear implant performance in geriatric patients. Laryngoscope.

[34] Looi V, She JHK (2010). Music perception of cochlear implant users: a questionnaire, and its implications for a music training program. Int J Audiol.

[35] Nemer JS, Kohlberg GD, Mancuso DM, Griffin BM, Certo MV, Chen SY, Chun MB, Spitzer JB, Lalwani AK (2016). Reduction of the harmonic series influences musical enjoyment with cochlear implants. Otol Neurotol.

[36] Eskridge EN, Galvin JJ, Aronoff JM, Li T, Fu Q (2012). Speech perception with music maskers by cochlear implant users and normal hearing listeners. J Speech Lang Hear Res.

[37] Drennan WR, Rubinstein JT (2008). Music perception in cochlear implant users and its relationship with psychophysical capabilities. J Rehabil Res Dev.

